# Length of stay following elective craniotomy for tumor resection in children and young adults: a retrospective case series

**DOI:** 10.1007/s11060-024-04887-w

**Published:** 2024-11-29

**Authors:** Emal Lesha, Jordan T. Roach, L. Erin Miller, C. Stewart Nichols, Brandy Vaughn, David G. Laird, Taylor Orr, Delaney Graham, Mustafa Motiwala, Nir Shimony, Paul Klimo

**Affiliations:** 1 Department of Neurosurgery, University of Tennessee Health Science Center, Memphis, TN USA; 2 Semmes Murphey, 6325 Humphreys Blvd, Memphis, TN 38120 USA; 3 St. Jude Children’s Research Hospital, Memphis, TN USA; 4 Atrium Health Carolinas Medical Center, Charlotte, NC USA; 5 College of Medicine, University of Tennessee Health Science Center, Memphis, TN USA; 6 Neuroscience Institute, Le Bonheur Children’s Hospital, Memphis, TN USA

**Keywords:** Case series, Brain tumor, Extended, Length of stay, Pediatric, Predictors

## Abstract

**Purpose:**

Length of stay (LOS) is a critical metric of healthcare delivery. Prolonged LOS is associated with a heightened risk of adverse complications. We aimed to provide a comprehensive evaluation of LOS, specifically identifying variables associated with extended LOS (eLOS), in children and young adults following elective craniotomy for tumor resection.

**Methods:**

All elective craniotomies for tumor resection performed at our tertiary care children’s hospital from January 2010 to December 2022 were included for review, excluding patients > 21 years of age. Demographic, clinical, and procedural variables for each craniotomy were collected. LOS was defined as the interval in days from index surgery to discharge. eLOS was defined as greater than 7 days.

**Results:**

1,276 patients underwent a total of 1,497 elective craniotomies for tumor resection. The median age was 9.45 years old, with the most common age group being > 10 years (45.6%). Most patients had supratentorial tumors (63.4%) and underwent de novo surgery (60.7%). Patients with an eLOS experienced longer ICU admissions, longer surgical times, and were younger. Variables found to be significantly associated with eLOS were posterior fossa resection (OR = 2.45), de novo craniotomy (OR = 0.49), prior shunt or ETV (OR = 1.80), tumor type (craniopharyngioma (OR = 3.74) and medulloblastoma (OR = 0.51)), and the presence of at least one postoperative event (POE) (OR = 29.85).

**Conclusion:**

This is the largest study evaluating factors (patient, tumor, surgical) associated with eLOS after elective craniotomy for tumor resection in children and young adults. The findings of this clinical study are important for preoperative counseling, neurosurgical team preparedness, and healthcare delivery optimization.

**Supplementary Information:**

The online version contains supplementary material available at 10.1007/s11060-024-04887-w.

## Introduction

Length of stay (LOS) is a critical metric of healthcare delivery. Prolonged LOS is associated with a heightened risk of adverse and unintended complications, such as surgical site and other hospital-acquired infections [1]. Moreover, longer hospitalization leads to increased financial and emotional burden for the healthcare system and patient, respectively. Analyzing LOS is, therefore, critical to lowering patient and system-level strain.

Several studies have identified factors that may predict protracted LOS after elective craniotomy for tumors. These include age, race, perioperative seizure, and postoperative complications, among others [2–5]. With many of these studies focusing predominantly on adults, a better understanding of factors affecting LOS in children and adolescents undergoing craniotomy for tumor resection is needed [6]. The adult and pediatric brain tumor populations differ substantially in many respects, such as tumor types, location, preexisting comorbidities and ability to recover from surgery. To this end, we aim to provide a comprehensive evaluation of LOS, with a particular focus on identifying variables associated with a prolonged LOS, in children and young adults following elective craniotomy for tumor resection.

## Methods

All craniotomies for tumor resection performed at the authors’ tertiary care children’s hospital are recorded in a prospectively maintained departmental database specifically designed for clinical research and quality improvement. All procedures performed from January 1, 2010, through December 31, 2022, were included. Demographic, clinical, and procedural variables for each elective craniotomy for tumor resection were collected. Patients > 21 years old undergoing tumor resections were excluded from the study. Craniotomies done on an urgent or emergent basis, or for reasons other than resection of tumor such as biopsy, fenestration or placement of catheter into tumor cyst(s), were excluded. Other non-craniotomy tumor operations such as transnasal/endonasal and laser ablation were likewise not included.

Patient, tumor and operative data points were collected for each admission. Age was categorized as infant/toddler (0–4 years), young child (5–9 years) and pre-teen/teen/young adult (10 years and older). Insurance status was either private or public/none. Although there were 3 surgeons (A-C) during the study period, 94% of the operations were performed by surgeons A and B. A patient with preexisting treated hydrocephalus was one who was dependent on a shunt or a patent third ventriculostomy at the time of their resection. Surgery was either de novo, meaning no prior craniotomy at that site, or a repeat/redo at the same site. Tumor grade was categorized as either high or low based on histologic and molecular analysis and according to the latest WHO classification of tumors. There were few intermediate grade tumors, and these were classified as high.

A postoperative event (POE) was defined as a notable medical or surgical occurrence, anticipated or unanticipated, that developed during the inpatient postoperative period, resulting in further testing, evaluation, follow-up or intervention. POEs included all unanticipated post-procedural events, but also those that were anticipated or carried a high risk of occurrence (e.g., worse hemiparesis after resection of a brainstem tumor or diabetes insipidus after resection of a craniopharyngioma). Disposition was the place where the patient went after leaving the hospital. LOS was defined as the interval from the date of index surgery to the date of discharge. In the event that a patient underwent more than one elective craniotomy during the same admission, only the last surgery was included for analyses. Extended LOS (eLOS) was defined as greater than 7 days.

### Statistical analysis

Continuous variables are reported using median and interquartile range (IQR), while categorical variables are summarized with frequency and percentage. To assess the association of each variable with the dichotomous outcome of extended LOS, bivariate and multivariate analyses were conducted using generalized estimating equations with an exchangeable correlation to account for patients with multiple hospitalizations. The final multivariate model was obtained using a backward model selection procedure, which is an iterative process, retaining only variables with *p* < 0.05. Adjusted odds ratios and 95% confidence intervals (CI) were calculated based on these analyses. All analyses were conducted using SAS 9.4 (SAS Institute, Cary, NC).

This case series has been reported in line with the PROCESS Guideline [7].

## Results

**Fig. 1:**
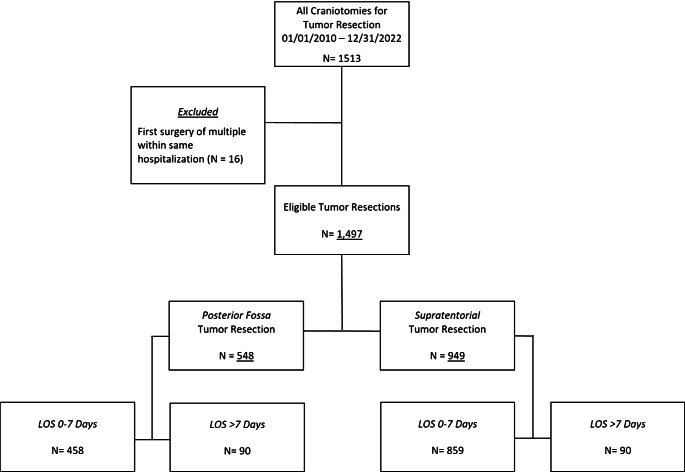
Study eligibility, inclusion criteria, and case distribution

1,276 patients underwent a total of 1,497 elective craniotomies for tumor resection during the study period (Fig. 1). Only 52 encounters (3.5%) included a second elective craniotomy during the same hospitalization. As shown in Tables 1 and 2, 58.5% of the operations were performed in male patients, 65.6% in Caucasians and 76.6% of patients had private insurance. The median age at time of surgery was 9.45 years old (range, 1 mo–21 yo). The most common age group was > 10 years (45.6%) followed by < 5 years (27.2%) and 5–10 years of age (27.2%). The majority of patients had supratentorial tumors (63.4%), de novo surgery (i.e., not a repeat craniotomy at the same site, 60.7%), and no preexisting treated hydrocephalus (80.0%). A POE occurred in 36.3% of cases, 29.0% of which were surgical only, 13.8% medical only, and 13.7% both. The POE was unanticipated in 26% of cases.

**Table 1:** Encounter characteristics and patient demographics

Age (mean)	9.45 years old	
Race	Caucasian	982 (65.6%)
	African American	210 (14.0%)
	Hispanic/Asian/Other	305 (20.4%)
Sex	Female	621 (41.5%)
	Male	876 (58.5%)
Health Insurance	Private	1147 (76.6%)
	Public/none	350 (23.4%)
Tumor Location	Supratentorial	949 (63.4%)
	Infratentorial	548 (36.6%)
Length of stay (mean, range)	4.82 days (1–60 days)	
ICU stay (mean, range)	2.25 days (0–37 days)	
Surgical time (mean, range)	266.96 min (30–743 min)	

Overall, the 1,497 hospitalizations had a median LOS of 4.82 days (IQR 0.37- 60 days), 180 (12.0%) of which were associated with an eLOS. Over the duration of the study period (13 years), for the annual variability of both average LOS (4.8–6.2 days) (Online Resource 1) and proportion of eLOS (6.2-21.7%) (Online Resource 2), there has been less variability and a general downward trend with time for both.

Table 2 summarizes patient and tumor characteristics by LOS. Only 46 of these hospitalizations resulted in admission to inpatient rehabilitation, 38 of which belonged to the eLOS group (82.6%); the remaining patients were discharged home. Patients with an eLOS had on average longer ICU admissions (7.59 days vs. 2.25 days), longer surgical times (355.12 min vs. 266.96 min), and were younger in age (8.63 years vs. 9.61 years) (Online Resource 3). Online Resource 4 compares patients based on craniotomy location (infra- vs. supratentorial). Patients who underwent posterior fossa craniotomies had, on average, longer surgical times (287.2 min vs. 255.28 min), longer LOS (5.84 days vs. 4.82 days), longer ICU stays (2.74 days vs. 2.25 days), and were younger in age (8.4 years vs. 10.05 years). Data on LOS by type and grade of tumor are represented in Online Resource 5. Patients undergoing resection for craniopharyngioma and ependymoma had the highest rates of eLOS at 22.1% and 15.6%, respectively; those undergoing resections for high grade glioma had the lowest rates of eLOS at 7.1%.

**Table 2:** Encounter characteristics between standard and extended LOS patients

Standard vs. Extended LOS (*N* = 1497)		0–7 days	> 7 days
		Number of encounters	
Age	0 to 4 years	277	56
	5 to 9 years	349	48
	10 + years	689	76
Race	Caucasian	870	112
	African American	180	30
	Hispanic/Asian/Other	267	38
Sex	Male	769	107
	Female	548	73
Health Insurance	Private/St. Jude	1012	135
	Medicaid/Medicare or none	303	47
Surgeon	A	602	89
	B	636	79
	C	79	12
Prior ETV or Shunt	Existing Shunt	185	39
	Prior ETV	79	15
	Prior Shunt or ETV	264	54
Prior Craniotomy	No + Yes (different site)	782	125
	Yes (same site)	535	54
Tumor Grade	High	625	75
	Low	692	105
Tumor Type	Medulloblastoma	181	20
	Ependymoma	168	31
	Craniopharyngioma	88	25
	Other	158	17
	Germ cell tumors	24	3
	Low grade gliomas	451	59
	High grade gliomas	131	10
	Embryonal tumors	116	15
Postoperative Event (POE)	Yes	379	164
Disposition	Home/Local housing	1291	137
	Inpatient rehab	10	36
	Unknown	16	7

Reasons for an eLOS were multiple, but most commonly included a POE (164/180, 91.1%): surgical (49.4%), medical (32.8%), or both (10.6%). Only 9.4% of patients with an eLOS required return to the operating room for an additional procedure. Similarly, 10.0% of eLOS were due to awaiting placement to an inpatient facility and only 1.1% while awaiting authorization for outpatient services.

Table 3 summarizes the results of the bivariate analysis, which tested the association between each variable and eLOS. Significant variables included age < 5 years (*p* = 0.002), the presence of an existing shunt or ETV (*p* = 0.01), de novo craniotomy (*p* = 0.01), posterior fossa resection (*p* < 0.001), tumor type (*p* = 0.03), and the presence of at least one POE (*p* < 0.001). On multivariate analysis (Table 4), variables that were found to be significantly associated with eLOS were: posterior fossa resection (OR = 2.45; 95% CI 1.61–3.74; *p* < 0.001), de novo craniotomy (OR = 0.49; 95% CI 0.31–0.76; *p* = 0.002), prior shunt or ETV (OR = 1.80; 95% CI 1.07–3.03; *p* = 0.027), tumor type (in particular craniopharyngioma (OR = 3.74; *p* = 0.001) and medulloblastoma (OR = 0.51; *p* = 0.04) when compared to low grade gliomas), and the presence of at least one POE (OR = 29.85; 95% CI 14.92–59.74; *p* < 0.001).

**Table 3:** Bivariate analysis results utilized to determine association between each variable and extended LOS

Bivariate analysis	OR estimate	95% CI		*p*-value
Age (overall)	0.97	0.94	1.00	0.06
Age (categorical)				
0 to 4 years	1.83	1.24	2.70	*0.002*
5 to 9 years	1.25	0.84	1.84	0.27
10 + years	Reference			
Race				
African American	1.30	0.81	2.07	0.28
Hispanic/Asian/Other	1.11	0.74	1.66	0.63
Caucasian	Reference			
Sex	0.96	0.69	1.33	0.79
Health Insurance	0.85	0.59	1.22	0.38
Surgeon				
A	0.97	0.49	1.92	0.94
B	0.82	0.41	1.62	0.56
C	Reference			
Prior ETV or Shunt	1.69	1.14	2.52	*0.01*
De Novo Craniotomy vs. Redo Craniotomy	0.63	0.45	0.90	*0.01*
Posterior fossa vs. Supratentorial tumor resection	1.88	1.36	2.59	*< 0.001*
Tumor grade	0.79	0.57	1.10	0.79
Tumor Type				*0.0336*
Craniopharyngioma	2.17	1.24	3.82	*0.007*
Embryonal tumors	0.99	0.52	1.86	0.97
Ependymoma	1.41	0.88	2.27	0.16
Germ cell tumors	0.96	0.27	3.33	0.94
High grade gliomas	0.58	0.28	1.24	0.16
Medulloblastoma	0.85	0.49	1.45	0.54
Other	0.82	0.46	1.48	0.52
Low grade gliomas	Reference			
Postoperative Event	27.68	14.81	51.75	*< 0.001*

**Table 4:** Multivariate analysis results utilized to determine association between each variable and extended LOS

Multivariate analysis	OR estimate	95% CI		*p*-value
Age (categorical)				
0 to 4 years	1.60	0.98	2.59	0.06
5 to 9 years	1.48	0.95	2.31	0.08
10 + years	Reference			
Prior ETV or Shunt	1.80	1.07	3.03	*0.027*
De Novo Craniotomy vs. Redo Craniotomy	0.49	0.31	0.76	*0.002*
Infratentorial vs. Supratentorial Tumor Resection	2.45	1.61	3.74	*< 0.001*
Tumor Type				
Craniopharyngioma	3.74	1.97	7.10	*< 0.001*
Embryonal tumors	0.58	0.26	1.30	0.19
Ependymoma	1.18	0.66	2.11	0.59
Germ cell tumors	1.41	0.36	5.46	0.62
High grade gliomas	0.63	0.30	1.29	0.21
Medulloblastoma	0.51	0.27	0.96	*0.04*
Other	0.84	0.46	1.55	0.58
Low grade gliomas	Reference			
Postoperative Event	29.85	14.92	59.74	*< 0.001*

## Discussion

### Our results

LOS has become an important hospital-based measure, particularly for high-risk, high-cost procedures such as craniotomy for tumor resection. Missios and Bekelis showed that LOS was a major contributor to cost changes in patients undergoing craniotomies for tumor resection [8]. There are few pediatric studies like ours. Hasan et al. evaluated LOS in newly diagnosed pediatric brain tumor patients 18 years of age and younger and showed that multiple surgeries, tumor location, subtotal resection, and posterior fossa syndrome were some of the variables associated with an extended LOS [6]. Mishra et al. found sodium imbalances, return to OR, postoperative pulmonary complications and massive blood loss during surgery were associated with an extended LOS [9].

In our study of almost 1500 elective tumor resections, the median LOS was 4.8 days. Only 180 admissions had an eLOS (12%). Not surprisingly, those patients that had an eLOS had longer ICU stays (7.59 vs. 2.25 days). Over 80% of patients that required inpatient rehabilitation had an eLOS. In their study, Hasan et al. defined eLOS as greater than 20 days, and had a 22.5% rate of eLOS [6]. In the adult population, Dasenbrock et al. studied LOS in over 11,000 patients using the National Surgical Quality Improvement Program, and found the median LOS to be 4 days, with 22.7% of patients having a LOS greater than 8 days [10].

Multivariable analysis found that preexisting hydrocephalus (shunt or ETV), infratentorial tumor location, tumor type, de novo craniotomy, and the occurrence of one or more POE were independently associated with eLOS. Each of these will be discussed in further detail. All other variables, including age, race, health insurance status, surgeon, and tumor grade were not significant.

### Preexisting treated hydrocephalus

The dependance on a shunt or third ventriculostomy increased the odds of an eLOS by 80%. This is not surprising as many children with brain tumors have hydrocephalus, especially those tumors that are intra- or paraventricular [11–12]. This finding is valuable when counseling patients and families preoperatively.

A prior study found the presence of an existing shunt to be significantly associated with eLOS in children undergoing an elective craniotomy of any kind (OR = 1.8) [13]. Shi et al. found the presence of preoperative shunt to be significantly associated with the risk of post-craniotomy intracranial infection in tumor patients [14]. We surmise that patients with preexisting hydrocephalus are, in general, a more complex population. More often they require longer inpatient monitoring and are at greater risk for POEs (48.8% vs. 33.6%). Common postoperative symptoms such as nausea, vomiting, headaches, and fevers may result in shunt evaluation (e.g., imaging, shunt tap). Any intradural, particularly intraventricular, surgery can result in a shunt malfunction due to blood products or debris within the CSF or bacterial contamination. The shunt failure risk is approximately 11% within 90 days of elective intradural surgery [15].

### Craniotomy history & location

De novo craniotomy (i.e., first-time operation or craniotomy at a new site) and tumor location were both associated with eLOS. De novo surgery lowered the odds by half. Conversely, a redo craniotomy doubled the odds of an eLOS. Similarly, an infratentorial tumor location increased the odds by 2.45 times. These associations are understandable. A redo craniotomy is often more difficult because of the altered surgical planes and anatomy, which can lead to a more difficult dissection, longer operative time, increased risk of complications, and ultimately longer postoperative recovery.

Posterior fossa surgery comes with possible issues that are either unique or more common compared to supratentorial surgery, such as pseudomeningoceles, hydrocephalus, cranial neuropathies, greater pain, and posterior fossa syndrome (PFS), all of which can result in greater LOS [16–17]. For example, Hasan et al. showed that PFS was a major predictor of extended LOS [6]. Post-resection hydrocephalus is present in 10–40% of children with posterior fossa tumors [12]. In our study, 45.2% of infratentorial tumor resections had some form of surgical POE, as compared to 35.2% of supratentorial resections.

### Tumor type

Tumor type, specifically craniopharyngioma or medulloblastoma, when compared to low grade gliomas as the reference group was found to be independently associated with eLOS. There was an increased odds of 3.74 times with resection of a craniopharyngioma. It is well recognized that craniopharyngiomas are one of the most challenging tumors to resect because of their supra-/parasellar location. The risk and variety of postoperative issues is significant and numerous, respectively [18–19]. Many of these patients have had one or more prior resection attempts, with preexisting neurologic, endocrine, hypothalamic and cognitive deficits. Monitoring for and managing diabetes insipidus, particularly when it is a new diagnosis in the postoperative period, adds at least several days to the ICU and overall hospital LOS [20].

Medulloblastoma, on the other hand, lowered the odds of an eLOS by 50%. This finding was rather unexpected. Patients with medulloblastomas are often young, with the most common location being in the 4th ventricle, and have accompanying obstructive hydrocephalus. These patients are at the highest risk of having PFS [21]. In addition, many of our medulloblastoma patients undergo redo or so-called “2nd look” surgery for suspected or unequivocal residual or recurrent tumor. Therefore, “medulloblastoma” being a protective covariate when compared to low grade gliomas is due, in part, to low risk of PFS and neurologic complications [22–23].

### POE

Of the 5 covariates identified as independent predictors of eLOS on multivariable analyses, the occurrence of a POE (medical, surgical or both) was, by far, the most impactful. Overall, 36.3% of all patients experienced a POE; however, 91.1% of patients with an eLOS experienced a POE compared to 28.8% of patients without an eLOS. A POE raised the odds of an eLOS by nearly an astonishing 30-fold.

Rates of postoperative complications for pediatric tumor craniotomies in the literature have been reported between 30 and 50% [24–26]. There is an abundance of literature looking at the interaction between postoperative complications and LOS for patients undergoing craniotomies, whether elective or emergent, but mainly in adult patients [2, 27–28]. Few studies have focused on children [6, 29–30]. A recent study found a nearly 15-fold increased odds of eLOS in children with one or more POE after an elective craniotomy of any kind [13]. In contrast to the other identified covariates in the current study, POE is, at least in part, modifiable. Our results strongly suggest that the attending surgeon recognize, to the best of their abilities, those patients at risk for POE. Preoperative risk analysis would hopefully allow some POEs to be prevented, while preparing for and promptly managing those that do occur, so as to lessen the burden and potential downstream sequalae.

### Strengths & limitations

The major strength of this study lies in the quality of the data, prospectively obtained by a single individual over more than a decade. When creating the database, we sought to capture key items, while keeping the burden of database maintenance manageable. With this retrospective analysis, more variables would have added greater depth to our analysis, such as preoperative neurologic status, tumor volume and proximity to critical neurovascular structures, and goal(s) of surgery. All of these can impact the estimated LOS, even in patients with the same tumor type. Additionally, one could argue that all POEs should be considered “complications”. However, we intentionally avoided the use of the word “complication” as all complications are a POE, but, in our opinion, not all POEs are considered a complication. This philosophy is similar to what was previously put forth by Landriel Ibañez et al. [31] and later applied by Ferroli et al. [32], although there is currently no agreed upon definition of complications in neurosurgery. While our definition of eLOS was rather arbitrary (as there is no universally accepted definition), we felt that 7 days was a reasonable period for an elective surgery. Because this is a single institution study, generalizability will naturally be limited. For example, our institution historically has a high volume of brain tumor patients and partners with a quaternary-level children’s cancer hospital, so our population characteristics will likely differ to some degree from other institutions.

## Conclusion

This is the largest study evaluating factors (patient, tumor, surgical) associated with eLOS after elective craniotomy for tumor resection in children and young adults. Five independent covariates were identified– preoperative treated hydrocephalus, tumor location, tumor type, craniotomy history, and POE. The occurrence of one or more POE (medical or surgical) had immense effect, increasing the odds of an eLOS by almost 30-fold. The findings of this clinical study are important for preoperative patient and family counseling, preparedness of the neurosurgical team, and optimization of healthcare delivery in this high-risk population.

## Electronic supplementary material

Below is the link to the electronic supplementary material.

Supplementary Material 1

## Data Availability

No datasets were generated or analysed during the current study.
