# Supplementary material for: Length of stay following elective craniotomy for tumor resection in children and young adults: a retrospective case series
Source: J Neurooncol. 2024 Nov 29;171(3):651–8. doi: 10.1007/s11060-024-04887-w (PMC11729059; doi:10.1007/s11060-024-04887-w)
Supplement: Supplementary file 1 — Supplementary Material 1 [file 11060_2024_4887_MOESM1_ESM.zip › NeuroOnc_1Supplementary Information.docx]

**Supplementary Information**

**Article Title:** Length of stay following elective craniotomy for tumor resection in children**:** a retrospective case series

**Journal name:** Journal of Neuro-Oncology

**Author names:** Emal Lesha, MD; Jordan T. Roach, MS; L. Erin Miller, MD; C. Stewart Nichols, BS; Brandy Vaughn, RN; David G Laird, BS; Taylor Orr, MD; Delaney Graham, BS; Mustafa Motiwala, MD; Nir Shimony, MD; Paul Klimo Jr., MD, MPH

**Corresponding author:**

Paul Klimo Jr., MD

Semmes Murphey

6325 Humphreys Blvd.

Memphis, TN 38120

Phone**:** (901) 522-7700

Email**:** [pklimo@semmes-murphey.com](mailto:pklimo@semmes-murphey.com)

C. Stewart Nichols, BS (ORCID 0009-0002-5810-1878)

Phone: (901) 488-0697

Email: [cnicho44@uthsc.edu](mailto:cnicho44@uthsc.edu)

**Supplementary Figures Captions**

**Online Resource 1** Average LOS by each year

**Online Resource 2** Rates of extended LOS by each year

**Online Resource 3**Analysis of length of stay by age of patient, surgical time and ICU duration

**Online Resource 4**Analysis of tumor location by LOS, age, operative time and ICU stay. PFTR=posterior fossa tumor resection; STR=supratentorial tumor resection.

**Online Resource 5** Extended LOS by tumor type
