# Supplementary figures and images for: Length of stay following elective craniotomy for tumor resection in children and young adults: a retrospective case series
Source: J Neurooncol. 2024 Nov 29;171(3):651–8. doi: 10.1007/s11060-024-04887-w (PMC11729059; doi:10.1007/s11060-024-04887-w)

## eLOS rates (%) vs. Year

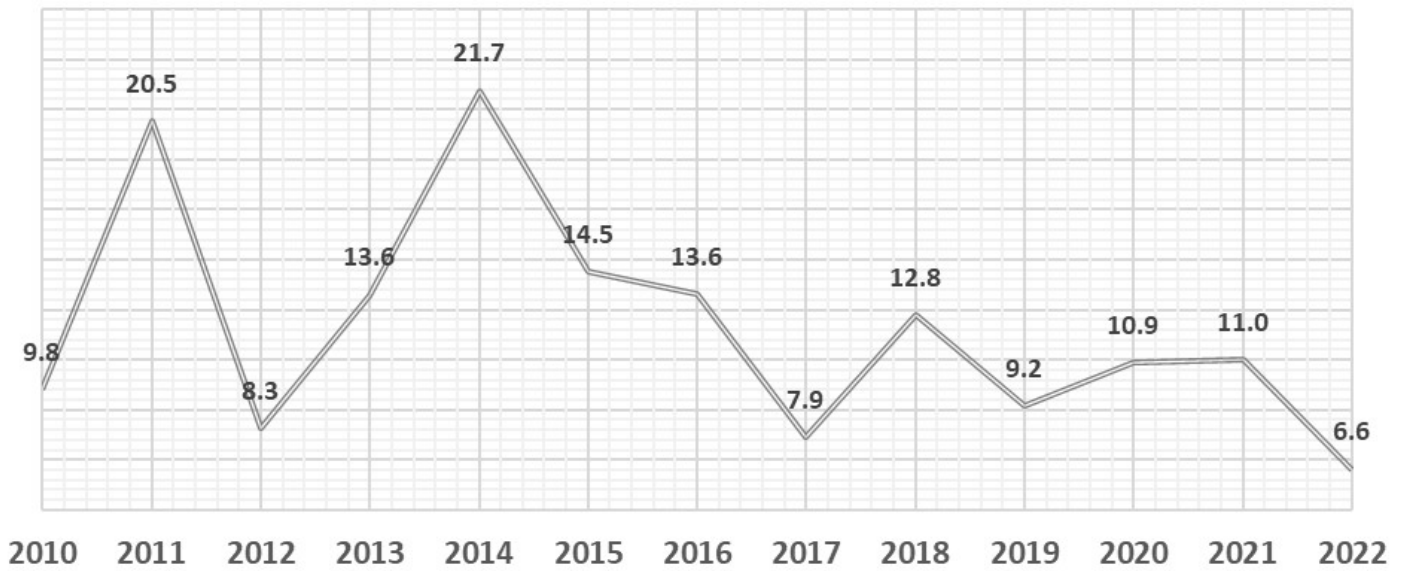

Supplement: Supplementary file 1 — Supplementary Material 1 [file 11060_2024_4887_MOESM1_ESM.zip › NeuroOnc_SM2.pdf]

**LOS vs. Age (years)**

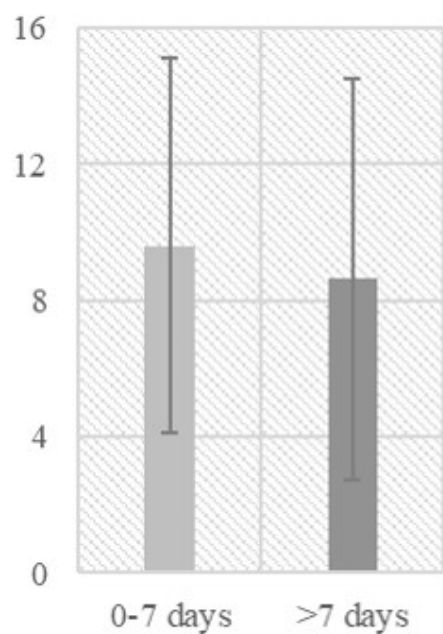

**LOS vs. OR Time (mins)**

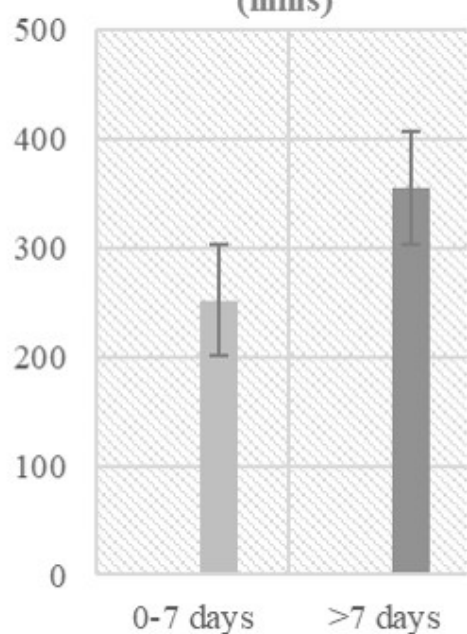

**LOS vs. ICU Time (days)**

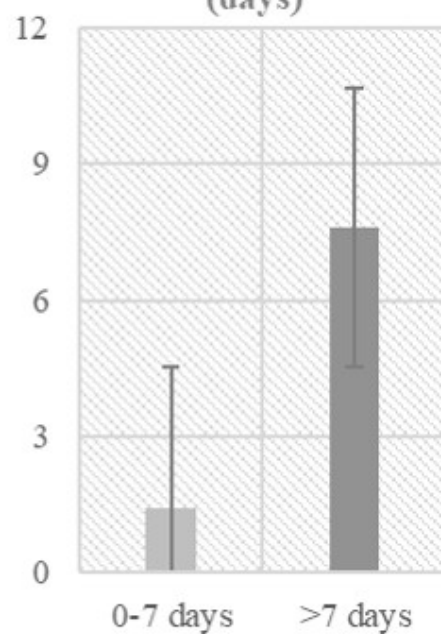

Supplement: Supplementary file 1 — Supplementary Material 1 [file 11060_2024_4887_MOESM1_ESM.zip › NeuroOnc_SM3.pdf]

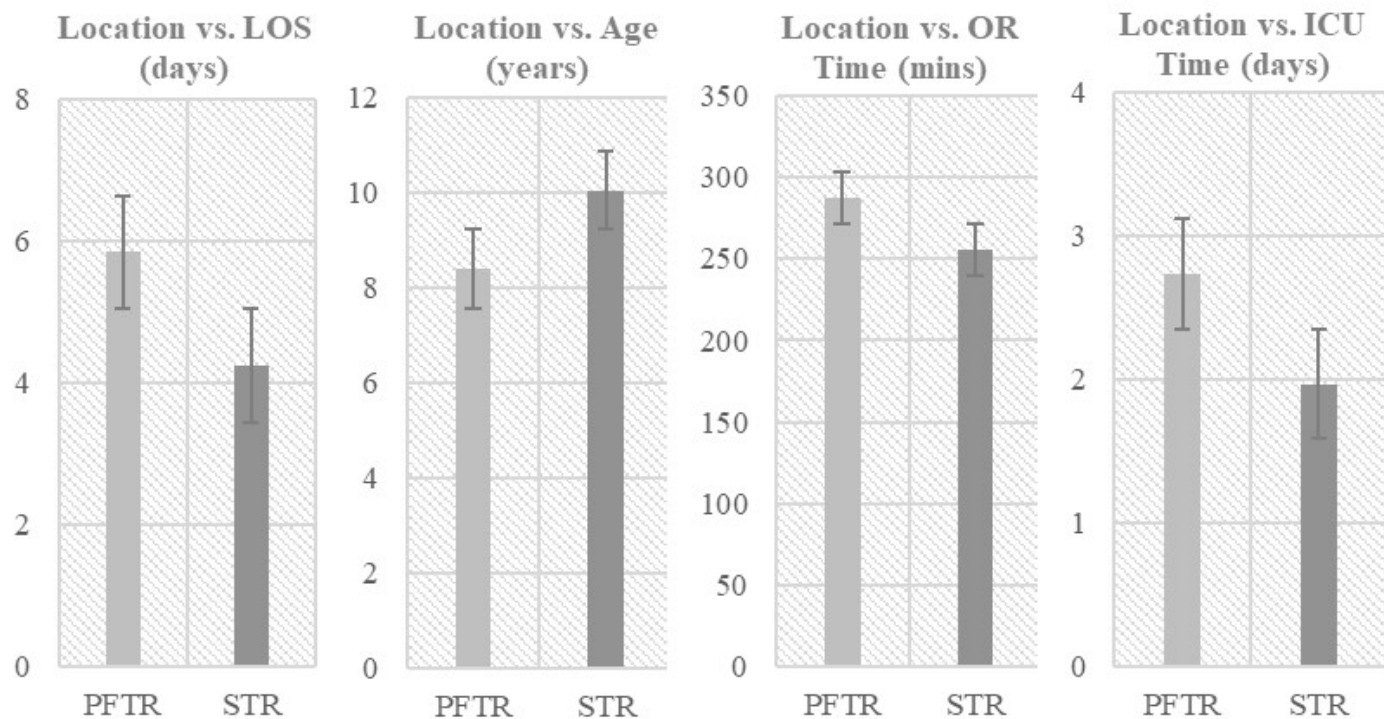

Supplement: Supplementary file 1 — Supplementary Material 1 [file 11060_2024_4887_MOESM1_ESM.zip › NeuroOnc_SM4.pdf]

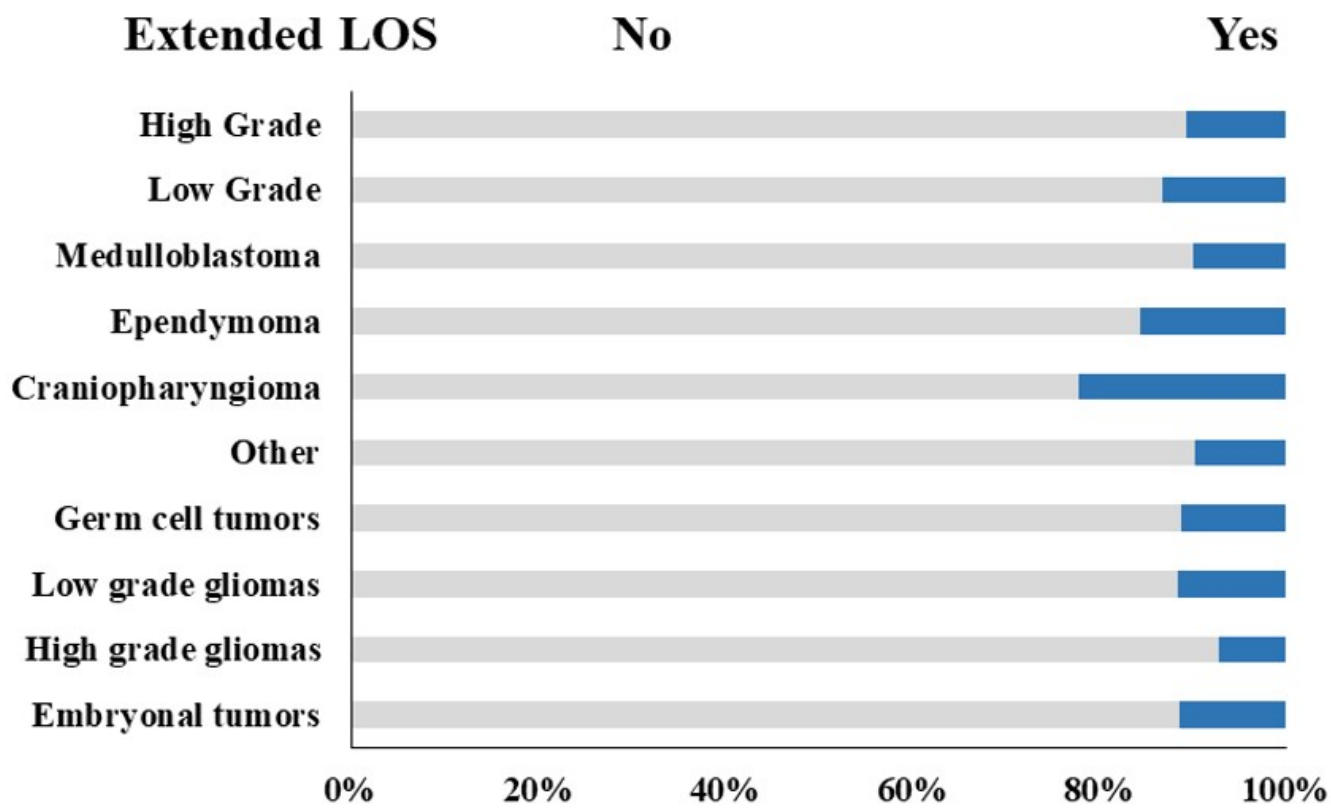

Supplement: Supplementary file 1 — Supplementary Material 1 [file 11060_2024_4887_MOESM1_ESM.zip › NeuroOnc_SM5.pdf]

## LOS (days) vs. Year

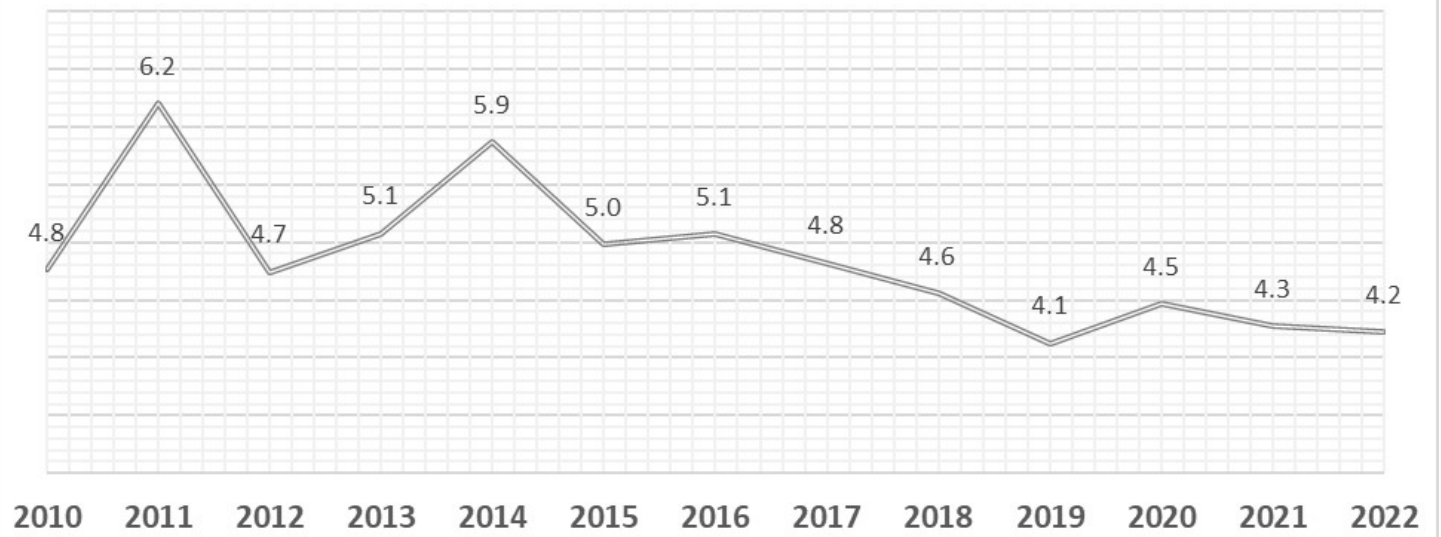

Supplement: Supplementary file 1 — Supplementary Material 1 [file 11060_2024_4887_MOESM1_ESM.zip › NeuroOnc_SM1.pdf]
